# UNDERSTANDING LONELINESS PATTERNS AMONG WIDOWED OLDER ADULTS OVER TIME AND THE PROTECTIVE EFFECT OF SOCIAL SUPPORT

**DOI:** 10.1093/geroni/igad104.2739

**Published:** 2023-12-21

**Authors:** Gina Lee, Daniel Russell

**Affiliations:** Iowa State University, Ames, Iowa, United States; Iowa State University, Ames, Iowa, United States

## Abstract

The present study aimed to investigate loneliness among widowed and non-widowed older adults. Additionally, the study sought to identify classes with different loneliness patterns among widowed older adults over time and to determine social resources that impact loneliness patterns after spousal loss. Data from the Health and Retirement Study were utilized to compare loneliness levels between widowed (n = 137) and non-widowed (n = 2,361). Those who were married at T1 (2008/2010), widowed at T2 (2012/2014), and remained widowed at T3 (2016/2018) were defined as widowed. T-tests were conducted to compare loneliness between the two groups, revealing that widowed individuals reported significantly higher levels of loneliness at T2 only. Using growth mixture models, three distinct loneliness patterns were identified among widowed individuals: Group 1, displaying mid-level loneliness at T1 and increasing over time (n=32); Group 2, exhibiting the lowest loneliness at T1 and leveling off (n=88); and Group 3, experiencing the highest loneliness at T1 and decreasing over time (n=17). Finally, analysis of variance tests were conducted to determine whether social support and engagement as measured at T1 differed among the three identified groups. Results indicated that social support from friends and children was significantly higher among group 2 compared to the other two groups. This study provides evidence of the protective effect of social support before widowhood on the psychological well-being of older adults after spousal loss. The findings may have implications for the development of interventions aimed at supporting widowed older adults during this difficult period of transition.

